# Predictors of Dietary Energy Density among Preschool Aged Children

**DOI:** 10.3390/nu10020178

**Published:** 2018-02-06

**Authors:** Nilmani N.T. Fernando, Karen J. Campbell, Sarah A. McNaughton, Miaobing Zheng, Kathleen E. Lacy

**Affiliations:** 1 School of Exercise and Nutrition Sciences, Deakin University, Burwood, VIC 3125, Australia; tnfernan@deakin.edu.au; 2 Institute for Physical Activity and Nutrition (IPAN), School of Exercise and Nutrition Sciences, Deakin University, Geelong, VIC 3220, Australia; karen.campbell@deakin.edu.au (K.J.C.); sarah.mcnaughton@deakin.edu.au (S.A.M.); j.zheng@deakin.edu.au (M.Z.)

**Keywords:** dietary energy density, preschool children, dietary intake, home food availability, non-core snacks, energy dense foods, Australia, 24-h recall

## Abstract

Childhood obesity is a global problem with many contributing factors including dietary energy density (DED). This paper aims to investigate potential predictors of DED among preschool aged children in Victoria, Australia. Secondary analysis of longitudinal data for 209 mother–child pairs from the Melbourne Infant Feeding, Activity and Nutrition Trial was conducted. Data for predictors (maternal child feeding and nutrition knowledge, maternal dietary intake, home food availability, socioeconomic status) were obtained through questionnaires completed by first-time mothers when children were aged 4 or 18 months. Three 24-h dietary recalls were completed when children were aged ~3.5 years. DED was calculated utilizing three methods: “food only”, “food and dairy beverages”, and “food and all beverages”. Linear regression analyses were conducted to identify associations between predictors and these three measures of children’s DED. Home availability of fruits (β: −0.82; 95% CI: −1.35, −0.29, *p* = 0.002 for DED_food_; β: −0.42; 95% CI: −0.82, −0.02, *p* = 0.041 for DED_food+dairy beverages_) and non-core snacks (β: 0.11; 95% CI: 0.02, 0.20, *p* = 0.016 for DED_food_; β: 0.09; 95% CI: 0.02, 0.15, *p* = 0.010 for DED_food+dairy beverages_) were significantly associated with two of the three DED measures. Providing fruit at home early in a child’s life may encourage the establishment of healthful eating behaviors that could promote a diet that is lower in energy density later in life. Home availability of non-core snacks is likely to increase the energy density of preschool children’s diets, supporting the proposition that non-core snack availability at home should be limited.

## 1. Introduction

Childhood obesity is a global problem. The World Health Organization estimated that 42 million children aged five years and under were overweight or obese in 2015, and these rates are expected to rise to 70 million by 2025 if current trends continue [1]. In Australia, approximately one in five children aged between two and four years is classified as overweight or obese [2]. Childhood obesity is associated with wide-ranging health and social risks [3] and it tends to track into adolescence and adulthood, with additional risks of negative health outcomes [3,4].

While the etiology of obesity is complex, energy intakes above energy requirements are a key contributor. One aspect of diet that may be particularly important is dietary energy density (DED), which is the concentration of energy in food or food and selected beverages (kJ/g). A systematic review by the 2010 Dietary Guidelines Advisory Committee in the United States (US) concluded that there was moderately strong evidence from longitudinal cohort studies in children and adolescents of a positive association between DED and increased adiposity [5]. Further, a 2016 meta-analysis demonstrated that DED was directly associated with the risk of excess adiposity among children and adolescents [6]. Short-term experimental feeding studies using covert adjustments of energy density demonstrated that energy density influences young children’s energy intake [7,8,9,10]. Additionally, nationally representative data in the US and large population surveys of children across several European countries showed that young children who consume diets that are lower in energy density have more healthful diets (less fat and sugar; more vegetables and fruits) than peers who consume diets that are higher in energy density [11,12].

The energy density of foods and beverages is influenced by their water content and macronutrient composition. Foods high in water and/or fiber tend to be low in energy density, while foods high in fat tend to be high in energy density. Beverages are generally low in energy density because of their high water content. There are different methods that can be used to calculate DED in order to adjust for the influence of beverages because beverages may disproportionately influence DED values [13]. While there is no consensus on which DED calculation method is most appropriate, calculating DED based on ‘food only’ is recommended for studies examining relationships with body weight [14]. Dairy beverages are part of a healthy diet as recommended by the Australian Dietary Guidelines [15] and provide substantial (12.5%) energy in two- to three-year-old children’s diets [16]. Given this, it is likely to be important to consider dairy beverages (or dairy alternatives) when assessing DED in young children’s diets [17,18].

With increasing evidence indicating that DED influences children’s body weight and energy intakes, it is important to identify the predictors of DED in order to better target and inform childhood obesity prevention interventions. Young children’s diets develop primarily within the family unit, making it vital to examine how family and demographic characteristics may influence DED [19]. The “Ecological model of predictors of childhood overweight” proposed by Davison and Birch in 2001 [20] is useful for identifying family and demographic characteristics as potential predictors of children’s DED. Several potential predictors, including parental nutrition knowledge [21], parental dietary intake [22], home food availability [23], and socioeconomic status [24], have been shown to be associated with various aspects of children’s diets; however, little is known about the predictors of children’s overall DED [11,12,25]. This study aimed to identify the predictors of DED among a sample of Australian preschool children using age-appropriate DED calculation methods.

## 2. Materials and Methods

### 2.1. Study Design and Participants

This study was a secondary analysis of data from the intervention and control participants in the Melbourne Infant Feeding, Activity and Nutrition Trial (Melbourne InFANT Program). The Melbourne InFANT Program was a cluster randomized controlled trial designed to test an intervention, where first-time parents were educated by experienced dietitians on how to promote healthy eating and active play in early childhood [26]. The intervention began when the children were 4 months of age and lasted until children were 18 months of age, with further data collection occurring at approximate ages 3.5 and 5 years [27]. A total of 542 families were recruited through Maternal and Child Health (MCH) centers within a 60-km radius from Deakin University, Melbourne, Australia [28]. Participants were equally and randomly assigned to either the intervention or control arm. Data were collected from participants who were English speaking and who provided informed consent. Parents were asked to complete questionnaires about their children and themselves at ages 4, 9, and 18 months and 3.5 and 5 years. Parents were also asked to provide three five-pass 24-h dietary recalls administered by telephone interview to record children’s dietary intakes when children were aged 9 and 18 months [26] and again at 3.5 and 5 years [27]. Dietary recalls were collected by dietitians on three non-consecutive days including a weekend day. Visual aids were provided prior to the interviews to help parents to determine food portion sizes. This is a valid and feasible method to collect dietary data [29,30,31]. The primary outcomes of the Melbourne InFANT Program are reported elsewhere [28]. Ethics approval was provided by Deakin University Ethics Committee (EC 175-2007) and by the Victorian Office for Children (CDF/07/1138). The current study utilized questionnaire data when children were 4 and 18 months and 3.5 years of age as well as the 24-h dietary recall data when children were 3.5 years of age.

### 2.2. Outcome Measure: Dietary Energy Density

Dietary energy density was assessed using 24-h dietary recall data [13,17] collected when children were approximately 3.5 years of age. Dietary intake data were coded by matching food and beverage items to their nutrient composition using the Australian Food Supplement and Nutrient Database “AUSNUT 2007” which has 4225 food and beverage items [32]. Additionally, the nutrient composition of items not found in the AUSTNUT 2007 database was included and classified according to information from the manufacturing company or the product’s nutrition information panel [28].

Dietary energy density was calculated using multiple age-appropriate methods including beverages most likely to be consumed by preschool aged children; food only (no beverages; DED_food_), food and dairy beverages (DED_food+dairy beverages_) and food and all beverages (included water; DED_food+all beverages_). Specific AUSNUT 2007 food groups were chosen for the DED_food_ and DED_food+all beverages_ variable calculations considering previously published criteria [13,33]. While previous studies have used a DED variable for ‘food and milk’ [17,18], the variable used in the present study, DED_food+dairy beverages_, included all dairy and dairy-substitute based beverages (e.g., soy, rice, oat beverages), dairy beverage flavorings, infant/toddler formula and breastmilk. More detailed information can be found in Appendix A Table A1, which includes details of the AUSNUT 2007 food codes included and excluded. Energy densities were calculated by dividing the kilojoules consumed by the weight of food (and beverages for DED_food+dairy beverages_ and DED_food+all beverages_) consumed using only the items relevant to each DED variable. As three dietary recall interviews were conducted for each child, daily DED values were calculated and averaged across the recalls.

### 2.3. Predictor Measures

The potential predictors of DED informed by the ecological model of predictors of childhood overweight [20] included: maternal child feeding and nutrition knowledge, maternal dietary intake, home food availability, and socioeconomic status where maternal education was used as a proxy. Maternal child feeding and nutrition knowledge, maternal dietary intake and home food availability were assessed at child age 18 months and maternal education was assessed at child age 4 months but was considered to be a proxy for socioeconomic status at child age 18 months. Table 1 lists the measures and their response options from the Melbourne InFANT Program questionnaires related to the different predictors that were analyzed, alongside the original sources of the measures.

Maternal knowledge of child feeding and nutrition was assessed using 12 questions related to the child feeding and nutrition intervention messages that were promoted in the Melbourne InFANT Program. Using methods similar to Spence et al. [34], correct answers were given a score of one and incorrect responses a score of zero and an overall summary score was calculated with a maximum score of 12. The total score for the 12 items was shown to have an intraclass correlation of 0.73 in test–retest assessments using a separate sample of 51 mothers with children aged approximately 18 months. Because total scores were non-normally distributed, a new dichotomous variable was created grouping mothers scoring at least 11 or scoring less than 11.

Maternal dietary intake was measured using the Cancer Council Victoria food frequency questionnaire (FFQ), Version 3 [35]. The questions included 98 foods and beverages, with responses ranging from ‘never’ to ‘three + per day’. For the present study, only intakes of fruits, vegetables, non-core sweet snacks, non-core savory snacks, and non-core drinks were analyzed because these groups represent major sources of foods in children’s diets and capture both ends of the energy density continuum. New dichotomous variables were created for each of the food groups using methods similar to those of Spence et al. [34]. Fruit consumption was dichotomized as per the Australian Dietary Guidelines [15], with mothers who reported consuming two or more servings per day grouped together and those consuming less than two serves per day grouped together. As few mothers met the Australian Dietary Guideline for vegetables (five servings per day) [15], vegetable consumption was dichotomized at three or more servings per day. Non-core sweet snacks consumption was dichotomized at one or more servings per day and non-core savory snacks consumption was dichotomized at one or more servings per week. Non-core drinks consumption was dichotomized at one or more glasses per day.

Home food availability was measured using seven questions completed by mothers. Four variables were created representing fruits, vegetables, non-core snacks and non-core drinks using methods similar to those of Collins et al. [37]. Vegetable and fruit availability were dichotomized as ‘always’ and ‘never/sometimes/usually’ available. The composite variable for non-core snacks was a summary score of the three items on chocolates or lollies, potato chips or salty snacks and cakes/donuts/sweet biscuits and was treated as a continuous variable with a maximum score of nine. Similarly, the composite variable for non-core drinks was a summary score of the two items on fruit juice and soft/sweetened beverages and was treated as a continuous variable with a maximum score of six.

Maternal education level was self-reported using one question where mothers indicated their highest education level. A dichotomous variable was created grouping mothers with more than or less than university education [38].

### 2.4. Covariates

Covariates for this study included: child’s sex (data collected through questionnaires completed by child’s parent when child was aged 4 months) and child’s age (data collected for covariate through questionnaires completed by parent when the child was around 3.5 years of age). As data were from both the intervention and control arms of the Melbourne InFANT Program, treatment arm was also used as a covariate. These covariates were included in all bivariate and multivariable linear regression models. The DAGitty program, version 2.3 (http://www.dagitty.net/dags.html) was used to identify additional potential confounding factors for each predictor [39] and these were included in multivariable linear regression analyses as appropriate.

### 2.5. Statistical Analysis

A total of 261 parent–child pairs participated in data collection when children were approximately 3.5 years of age. Exclusion criteria were applied excluding pairs where the mother was not a first-time mother (8 pairs) and where children had fewer than three five-pass 24-h dietary recalls at 3.5 years (5 pairs). Participants were excluded for missing data on the potential predictors: maternal child feeding and nutrition knowledge (11 pairs), maternal dietary intake (6 pairs), home food availability (1 pair) and maternal education (0 pairs). Further, participants were excluded when children had fewer than three five-pass 24-h dietary recalls at 18 months (18 pairs). Participants whose average energy intake exceeded the average energy intake of 5329 ± 1219 kilojoules by three or more standard deviations (3 pairs) were excluded. After excluding participants according to the above criteria, the final sample was 209 mother–child pairs.

Using IBM SPSS Statistics (version 24, 2016, IBM-SPSS Inc., Armonk, NY, USA), descriptive analysis was conducted to assess participants’ characteristics. Bivariate linear regression models were performed to examine the association between individual predictors and DED with adjustment for covariates including child sex, child age and treatment arm. Multivariable regression models including the previously described potential confounders were conducted to assess independent effects of each predictor on DED. These regression models controlled for DED at child 18 months, thus ensuring any associations seen were not due to baseline diet. The three DED variables were used as continuous outcomes, and separate models were fitted for the three DED measures. STATA statistical software (version 14, 2015, Stat Corp., College Station, TX, USA) was used to perform bivariate and multivariable linear regressions specifying parent groups as random effects to account for clustering.

## 3. Results

### 3.1. Sample Characteristics

Overall, children had a mean age of 3.5 ± 0.2 years with a slightly higher percentage of girls compared with boys (51% vs. 49%) (Table 2). There were slightly more children from the intervention arm of the study compared with the control arm (51% vs. 49%). Children had an average BMI *z*-score of 0.71 ± 0.89 and the average dietary energy intake including fiber was 5266 ± 1110 kJ. Overall, there was an overrepresentation of mothers with university or higher degrees (64%) relative to mothers with less education (36%).

### 3.2. Bivariate and Multivariable Linear Regression Analysis

Bivariate linear regressions showed significant associations between DED_food_ and maternal intake of fruits and home availability of fruits and non-core snacks (Table 3). Maternal intake of fruits (β: −0.39; 95% CI: −0.69, −0.08; *p* = 0.012) and home availability of fruits (β: −0.82; 95% CI: −1.35, −0.29, *p* = 0.002) had inverse associations in both bivariate and multivariable analyses. Home availability of non-core snacks (β: 0.11; 95% CI: 0.02, 0.20, *p* = 0.016) was positively associated with children’s DED in both bivariate and multivariable analyses.

Linear regressions showed significant associations between DED_food+dairy beverages_ and home availability of fruits and non-core snacks and maternal education used as an indicator of socioeconomic status (Table 4). Home availability of fruits (β: −0.42; 95% CI: −0.82, −0.02, *p* = 0.041) and maternal education (β: −0.24; 95% CI: −0.48, −0.01; *p* = 0.044) were inversely associated with children’s DED in both bivariate and multivariable analyses. Home availability of non-core snacks (β: 0.09; 95% CI: 0.02, 0.15, *p* = 0.010) showed positive associations with children’s DED for both bivariate and multivariable analyses.

Home food availability of fruits and non-core snacks showed significant associations with children’s DED_food+all beverages_. (Table 5). When bivariate analyses were conducted, home availability of fruits was inversely associated with children’s DED and home availability of non-core snacks was positively associated with children’s DED. However, neither predictor remained significant in multivariable analysis.

## 4. Discussion

This study aimed to investigate potential predictors of DED among preschool aged children. There were several significant relationships between the potential predictors and the energy density variables but the most consistent relationships were those between home availability of fruits and non-core snacks with DED, with significant associations found for DED_food_ and DED_food+dairy beverages_. Mean DED values were slightly lower for children in the present study than those reported among preschool children in the US [17].

Home availability of non-core snacks was predictive of preschool aged children’s DED_food_ and DED_food+dairy beverages_ and there was an inverse association between home availability of fruits and DED_food_ and DED_food+dairy beverages_. Previous studies have shown the availability of non-core snacks to have a positive association with children’s intake of non-core snacks [43,44]. Systematic reviews of older children (4–12 years, 6–11 years and 6–18 years) and studies among preschool children have also shown positive associations between home availability of fruits and children’s fruit consumption [45,46,47,48]. Consuming non-core snacks is problematic as it can reduce children’s dietary quality by increasing their intakes of fats and sugars [49]. Children’s preferences for non-core foods could also lead to consumption of these foods over core foods when both are available at home [39]. Therefore, it may be important to covertly restrict the availability of non-core snacks in order to moderate DED and promote intakes of fruits and vegetables [50]. Parents should promote healthy home food environments by favoring core foods over non-core foods from early in a child’s life.

Maternal dietary intakes of vegetables, non-core sweet or savory snacks and non-core drinks did not predict children’s DED. One exception was maternal intake of fruits, which was inversely associated with children’s DED _food_. Several studies have identified positive associations between parents’ and children’s fruit and vegetable intakes [51,52,53]. The lack of significant results for maternal intake of vegetables, non-core sweet or savory snacks and non-core drinks and children’s DED could be due to differences in how dietary data were collected (three 24-h dietary records for children and food frequency questionnaires for the past 12 months in mothers). It could also be the case that the diet of the father may be important when it comes to some specific foods. Another study using data from the Melbourne InFANT Program found that fathers’ intakes of fruits, sweet snacks and sweetened beverages when children were 20 months of age were associated with children’s intakes of fruits, sweet snacks and sweetened beverages between 20 months and 3.5 years [54]. It is important to note that DED could be influenced by paternal factors, as studies have found that father’s dietary intake of non-core snacks influenced children’s consumption of non-core snacks [51,54]. Further studies should consider examining the association between paternal dietary intakes and children’s DED.

Maternal education was inversely associated with children’s DED_food+dairy beverages_, which is consistent with previous research demonstrating that children from lower socioeconomic positions tend to have less healthful diets [55] and children of mothers with higher maternal educational levels tend to consume lower quantities of non-core snacks and more healthy snacks [25]. Kant and Graubard found that the education level of the household head, but not household income, was related to DED_food_ (the only DED variable used) for a nationally representative sample of two- to five-year-old children in the US. This suggests family education level may be more important in determining food choices and, therefore, future intervention studies should target increasing education-related knowledge gaps in target populations [25]. Future research should consider using more than one proxy measure to examine socioeconomic backgrounds when examining children’s DED [56,57].

DED_food_ and DED_food+dairy beverages_ tended to have similar values and similar associations with the potential predictors compared with DED_food+all beverages_. It has been suggested that DED should be calculated without the inclusion of beverages (e.g., “food only”) when focusing on the risk of obesity, as beverages tend to dilute energy densities leading to misinterpretation of results [58,59,60]. Also, since beverages tend to have lower energy densities (higher water content than macronutrient content) and different levels of satiety in comparison with food items (higher macronutrient content than water content), including beverages in energy density calculations can have a strong effect on overall energy density values. It is also important for researchers to agree on which beverages to include/exclude in DED calculations when examining the diets of preschool aged children.

The present study has several strengths, including the use of longitudinal data to identify the associations between multiple early life predictors that could influence the DED of preschool aged children. Dietary energy densities were calculated using three five-pass 24-h dietary recalls, which is the method recommended as the gold standard [29]. Also, this study used multiple DED variables and previously published measures to obtain data on the potential predictors and careful consideration was given to the potential confounders included in multivariable linear regression models. Limitations of this study include that the majority of the Melbourne InFANT Program mothers were highly educated with high child feeding and nutrition knowledge, which may limit the generalizability of results. Further, the diversity of dietary patterns that exists among preschool children [61,62] may not have been captured in the present study. All measures were self- or parent-reported, which could potentially increase social desirability bias and the exclusion of mothers with missing data for the potential predictors may have influenced the statistical analysis. Only mothers’ diet was considered a predictor in these analyses, but other potentially important role models of eating, including fathers, grandparents, carers and siblings, could also be influential. Lastly, there may be other relevant predictors that were not included in the present study, such as food accessibility [20] and food availability at child care, that could influence young children’s DED.

## 5. Conclusions

Home availability of fruits and non-core snacks influenced the DED of preschool aged children. This suggests that providing fruit at home early in a child’s life may encourage the establishment of healthful eating behaviors that could promote a diet that is lower in energy density later in life. Children from homes where non-core snacks were readily available early in life were more likely to consume diets that were higher in energy density even when their mothers had relatively high nutrition knowledge and educational backgrounds. Mothers should be advised to limit the presence of non-core snacks at home early on in their child’s life.

## Figures and Tables

**Table 1:** List of measures and responses from the Melbourne Infant Feeding, Activity and Nutrition Trial (InFANT) Program questionnaires considered as predictors.

Predictor	Age Assessed	Measure	Response	Source
Parenting styles and family characteristics				
Maternal child feeding and nutrition knowledge	18 months	12 items examined mothers’ knowledge of the child feeding and nutrition messages that were promoted in the Melbourne InFANT Program	Responses: ‘strongly agree’, ‘agree’, ‘disagree‘ and ‘strongly disagree’	Purpose-designed items previously used by Spence et al. [34]
Maternal dietary intake	18 months	Consumption over the previous 12 months of 98 food and beverage items was assessed. Selected items were used in this study:	Responses from a ten-point scale covering monthly, weekly and daily consumption rates: ‘never’, ‘<1 per month’, ‘1–3 per month’, 1 per week’, ‘2 per week’, ‘3–4 per week’, ‘5–6 per week’, ‘1 per day’, ‘2 per day’ and ‘3 + per day’	Adapted from Cancer Council Victoria food frequency questionnaire [35]
		fruits		
		vegetables, other than potato		
		non-core sweet snacks (cakes, sweet biscuits, ice cream, chocolate, other confectionary)		
		non-core savoury snacks (non-wholemeal crackers, chips, crisps)		
		non-core drinks (fruit/soft/orange/other drinks)		
Home food availability	18 months	7 items assessed home availability of:	Responses: ‘never’, ‘sometimes’, ‘usually’, ‘always’	Adapted from Macfarlane et al. [36]
		fruits		
		vegetables other than potato,		
		chocolates or lollies		
		cakes/donuts/sweet biscuits		
		potato chips or salty snacks		
		fruit juice and soft/sweetened beverages		
Community, demographical and societal characteristics				
Socioeconomic status	4 months	What is the highest level of schooling you have completed?	Responses: year 10 or equivalent, year 12 or equivalent, trade/apprenticeship, certificate and diploma, university degree and higher university degree	

**Table 2:** Characteristics of children, mothers and homes in the Melbourne InFANT Program.

Child Characteristics	Values
Energy intake (kJ)	
Mean ± SD	5266 ± 1110
Range	3050–8752
Energy density—Food only (kJ/g)	
Mean ± SD	6.42 ± 1.18
Range	3.92–10.05
Energy density—Food and dairy beverages (kJ/g)	
Mean ± SD	5.35 ± 0.90
Range	3.43–8.45
Energy density—Food and all beverages (kJ/g)	
Mean ± SD	3.38 ± 0.71
Range	1.21–6.36
Child’s age (years)	
Mean ± SD	3.5 ± 0.2
Range	3.2–4.2
Child’s sex	
Male, *n* (%)	103 (49)
Female, *n* (%)	106 (51)
Treatment arm	
Intervention *n* (%)	107 (51)
Control *n* (%)	102 (49)
Body mass index *z* score	
Mean ± SD	0.71 ± 0.89
Range	−1.94–4.04
Maternal and home characteristics	
Maternal child feeding and nutrition knowledge assessed when the child was 18 months	
≥11 (possible score 12), *n* (%)	77 (37)
<11 (possible score 12), *n* (%)	132 (63)
Maternal dietary intake assessed when the child was 18 months	
Fruits	
≥2 servings/day, *n* (%)	114 (55)
<2 servings/day, *n* (%)	95 (45)
Vegetables	
≥3 servings/day, *n* (%)	111 (53)
<3 servings/day, *n* (%)	98 (47)
Non-core sweet snacks	
≥1 servings/day, *n* (%)	84 (40)
<1 servings/day, *n* (%)	125 (60)
Non-core savoury snacks	
≥1 servings/week, *n* (%)	105 (50)
<1 servings/week, *n* (%)	104 (50)
Non-core drinks	
≥1 servings/day, *n* (%)	72 (34)
<1 servings/day, *n* (%)	137 (66)
Home food availability assessed when child was 18 months	
Fruits	
Always, *n* (%)	190 (91)
Never/sometimes/usually, *n* (%)	19 (9)
Vegetables	
Always, *n* (%)	193 (92)
Never/sometimes/usually, *n* (%)	16 (8)
Non-core snacks (possible score from 0 to 9)	
Median (IQR)	3.0 (3.0, 4.0)
Range	0.0–9.0
Non-core drinks (possible score from 0 to 6)	
Median (IQR)	2.0 (2.0, 4.0)
Range	0.0–6.0
Maternal education assessed when the child was assessed at 4 months.	
Less than university, *n* (%)	75 (36)
University or higher degree, *n* (%)	134 (64)

**Table 3:** Results of bivariate and multivariable linear regression analyses among children participating in the Melbourne InFANT Program (*n* = 209) for the predictors and the dietary energy density variable dietary energy density from food (DED_food_).

Predictor	Bivariate Regression ^1^			Multivariable Regression ^1,2^		
	β Coefficient	95% Confidence Interval	*p* Value ^3^	β Coefficient	95% Confidence Interval	*p* Value
Parenting styles and family characteristics						
Maternal child feeding and nutrition knowledge assessed when the child was age 18 months	−0.277	−0.612–0.057	0.104	−0.127	−0.453–0.198	0.444
Maternal dietary intake assessed when the child was age 18 months						
Fruits (≥2 servings/day)	−0.476	−0.787–−0.166	**0.003**	−0.386	−0.688–−0.085	**0.012**
Vegetables (≥3 servings/day)	−0.097	−0.414–0.221	0.551	−0.026	−0.331–0.278	0.865
Non-core sweet snacks (≥1 serves/day)	0.312	−0.007–0.631	0.055	0.322	0.018–0.625	**0.038**
Non-core savoury snacks (≥1 serves/week)	0.106	−0.211–0.423	0.513	0.029	−0.274–0.333	0.849
Non-core drinks (≥1 servings/day)	0.190	−0.138–0.518	0.257	0.103	−0.212–0.418	0.520
Home food availability assessed when child was age 18 months						
Fruits	−1.015	−1.558–−0.472	**<0.001**	−0.823	−1.353–−0.293	**0.002**
Vegetables	−0.470	−1.057–0.118	0.117	−0.317	−0.878–0.244	0.268
Non-core snacks	0.139	0.048–0.230	**0.003**	0.109	0.021–0.196	**0.016**
Non-core drinks	0.059	−0.045–0.163	0.267	0.048	−0.052–0.147	0.347
Community, demographic and societal characteristics						
Socioeconomic status assessed when the child was age 4 months	−0.262	−0.593–0.068	0.120	−0.142	−0.460–0.177	0.384

^1^ all bivariate and multivariable regression models included child sex and age, treatment arm and clustering by first-time parent group; ^2^ all multivariable regression models included children’s DED_food_ at child age 18 months. The model for the predictor maternal child feeding and nutrition knowledge was adjusted for maternal education [40]. The models for maternal dietary intake predictors were adjusted for maternal child feeding and nutrition knowledge and maternal education [41]. The models for home food availability predictors were adjusted for maternal child feeding and nutrition knowledge and maternal education [42]. The model for predictor maternal education had no additional adjustments; ^3^ all bolded results show significant associations (*p* ≤ 0.05).

**Table 4:** Results of bivariate and multivariable linear regression analyses among children participating in the Melbourne InFANT Program (*n* = 209) for the predictors and the dietary energy density variable dietary energy density from food and dairy beverages (DED_food+dairy beverages_).

Predictor	Bivariate Regression ^1^			Multivariable Regression ^1,2^		
	β Coefficient	95% Confidence Interval	*p* Value ^3^	β Coefficient	95% Confidence Interval	*p* Value
Parenting styles and family characteristics						
Maternal child feeding and nutrition knowledge assessed when the child was age 18 months	−0.219	−0.473–0.036	0.092	−0.113	−0.355–0.130	0.362
Maternal dietary intake assessed when the child was age 18 months						
Fruits (≥2 servings/day)	−0.207	−0.448–0.034	0.093	–0.135	−0.364–0.094	0.249
Vegetables (≥3 servings/day)	0.061	−0.183–0.305	0.624	0.129	−0.100–0.357	0.271
Non-core sweet snacks (≥1 serves/day)	0.231	−0.013–0.475	0.064	0.288	0.061–0.515	**0.013**
Non-core savoury snacks (≥1 serves/week)	0.183	−0.057–0.422	0.135	0.126	−0.100–0.352	0.275
Non-core drinks (≥1 serves/day)	0.252	0.001–0.503	**0.049**	0.185	−0.051–0.421	0.124
Home food availability assessed when child was age 18 months						
Fruits	−0.497	−0.921–−0.073	**0.022**	–0.417	−0.817–−0.017	**0.041**
Vegetables	−0.191	−0.643–0.260	0.406	–0.072	−0.494–0.349	0.737
Non-core snacks	0.109	0.040–0.179	**0.002**	0.086	0.021–0.152	**0.010**
Non-core drinks	0.037	−0.043–0.116	0.368	0.029	−0.046–0.104	0.447
Community, demographic and societal characteristics						
Socioeconomic status assessed when the child was age 4 months	−0.297	−0.548–−0.046	**0.020**	−0.244	−0.481–−0.007	**0.044**

^1^ all bivariate and multivariable regression models included child sex and age, treatment arm and clustering by first-time parent group; ^2^ all multivariable regression models included children’s DED_food+dairy beverages_ at child age 18 months. The model for the predictor maternal child feeding and nutrition knowledge was adjusted for maternal education [40]. The models for maternal dietary intake predictors were adjusted for maternal child feeding and nutrition knowledge and maternal education [41]. The models for home food availability predictors were adjusted for maternal child feeding and nutrition knowledge and maternal education [42]. The model for predictor maternal education had no additional adjustments; ^3^ all bolded results show significant associations (*p* ≤ 0.05).

**Table 5:** Results of bivariate and multivariable linear regression analyses among children participating in the Melbourne InFANT Program (*n* = 209) for the predictors and the dietary energy density variable dietary energy density from food and all beverages (DED_food+all beverages_).

Predictor	Bivariate Regression ^1^			Multivariable Regression ^1,2^		
	β Coefficient	95% Confidence Interval	*p* Value ^3^	β Coefficient	95% Confidence Interval	*p* Value
Parenting styles and family characteristics						
Maternal child feeding and nutrition knowledge assessed when the child was age 18 months	−0.039	−0.240–0.162	0.704	0.037	−0.148–0.223	0.694
Maternal dietary intake assessed when the child was age 18 months						
Fruits (≥2 servings/day)	−0.067	−0.256–0.123	0.490	−0.087	−0.262–0.087	0.327
Vegetables (≥3 servings/day)	−0.088	−0.278–0.102	0.365	−0.046	−0.221–0.130	0.610
Non-core sweet snacks (≥1 serves/day)	0.159	−0.032–0.351	0.103	0.068	−0.110–0.245	0.456
Non-core savoury snacks (≥1 serves/week)	0.048	−0.141–0.238	0.616	0.022	−0.152–0.195	0.806
Non-core drinks (≥1 serves/day)	−0.040	−0.237–0.157	0.694	−0.085	−0.265–0.096	0.356
Home food availability assessed when child was age 18 months						
Fruits	−0.413	−0.743–−0.082	**0.014**	−0.233	−0.544–0.079	0.143
Vegetables	−0.249	−0.601–0.104	0.166	−0.092	−0.416–0.233	0.579
Non-core snacks	0.084	0.030–0.139	**0.002**	0.046	−0.006–0.098	0.081
Non-core drinks	−0.003	−0.066–0.059	0.919	0.000	−0.057–0.057	0.990
Community, demographic and societal characteristics						
Socioeconomic status assessed when the child was age 4 months	0.014	−0.185–0.213	0.891	0.049	−0.132–0.229	0.598

^1^ all bivariate and multivariable regression models included child sex and age, treatment arm and clustering by first-time parent group; ^2^ all multivariable regression models included children’s DED_food+all beverages_ at child age 18 months. The model for the predictor maternal child feeding and nutrition knowledge was adjusted for maternal education [40]. The models for maternal dietary intake predictors were adjusted for maternal child feeding and nutrition knowledge and maternal education [41]. The models for home food availability predictors were adjusted for maternal child feeding and nutrition knowledge and maternal education [42]. The model for predictor maternal education had no additional adjustments; ^3^ all bolded results show significant associations (*p* ≤ 0.05).

## Appendix A

**Table A1:** The food groups that were included and excluded in calculating the three DED variables identified via the AUSTNUT 2007 two-digit, three-digit and five-digit food codes ^1,2^.

Variable	Definition	Austnut 2007 Food Codes
DED_food_	All solid and liquid based foods	12—Cereals and cereal products
		13—Cereal-based products and dishes
		14—Fats and oils
		15—Fish and seafood products and dishes
		16—Fruit products and dishes
		17—Egg products and dishes.
		18—Meat, poultry and game products and dishes
		21—Soup
		22—Seed and nut products and dishes (except food code 22203003—Coconut, fresh, mature, water or juice and food code 22204023—Almond milk, with linseed oil and water)
		23—Savory sauces and condiments
		24—Vegetable products and dishes
		25—Legume and pulse products and dishes
		26—Snack foods
		27—Sugar products and dishes (except food code 27303005—Ice confection drink, crushed ice with fruit-based flavored syrup (25% apple juice) and food code 27303006—Ice confection, 97% fruit juice, tropical (apple, mango, passionfruit & banana)
		28—Confectionery and cereal/nut/fruit/seed bars (except food code 28303—Chewing gum, Sugar sweetened and food code 28304—Chewing gum, Artificially sweetened)
		19109—Milk, evaporated, undiluted
		192—Yoghurt
		193—Cream
		194—Cheese
		195—Frozen milk products
		196—Custards
		197—Other dishes where milk is a major component
		20101003—Cream, soy
		203—Cheese substitute
		204—Soy-based ice confection
		205—Soy-based yoghurts
		31102—Yeast, vegetable and meat extracts
		31302011—Mustard, cream-style, condiment
		322—Infant cereal products
		323—Infant foods
		Excluded major codes were:
		11—Non-alcoholic beverages,
		30—Special dietary foods
		33—Dietary supplements.
DED_food + dairy beverages_	All solid and liquid foods including all dairy and dairy-substitute based beverages, dairy beverage flavorings, infant/toddler formula and breastmilk	All food codes included in DED_food_ are included with the below additional codes;
		11801—Fortified dry beverage flavorings
		11802—Fortified beverage flavorings made up, unspecified strength
		11803—Unfortified dry beverage flavorings
		11804—Unfortified beverage flavorings, made up, unspecified strength
		19101—Milk, cow, fluid, fat-increased
		19102—Milk, cow, fluid, regular whole, full fat
		19103—Milk, cow, fluid, regular whole, full fat, fortified
		19104—Milk, cow, fluid, reduced fat, <2%
		19105—Milk, cow, fluid, reduced fat, <2%, fortified
		19106—Milk, cow, fluid, skim, non-fat
		19107—Milk, cow, fluid, skim, non-fat, fortified
		19108—Milk, cow, fluid, added substances other than nutrients (e.g., Phytosterols)
		19112—Milk, powder, dry, skim
		19113—Milk, fluid, other (e.g., Goat and Sheep)
		19114—Milk, fluid, unspecified
		19801—Milk, other flavored and milk based drinks, full fat
		19802—Milk, coffee/chocolate flavored and milk-based drinks, full fat
		19803—Milk, other flavored and milk-based drinks, reduced fat
		19804—Milk, coffee/chocolate flavored and milk-based, reduced fat
		20101—Soy-based beverage, plain
		20102—Soy-based beverage, plain, fortified
		20103—Soy-based beverage, plain, reduced fat
		20104—Soy-based beverage, plain, reduced fat, fortified
		20105—Soy-based, plain, skim
		20106—Soy-based beverage, plain, skim, fortified
		20107—Rice—based beverage, plain
		20108—Oat-based beverage plain
		20202—Soy-based beverage, flavored, fortified
		20203—Soy-based beverage, reduced fat, flavored
		20204—Soy-based beverage, reduced fat, flavored, fortified
		22204023—Almond milk with linseed oil and water
		30102002—Breakfast cereal, beverage, all flavours, added vitamins A, B1, B2, C & folate
		30103—Milk-based powder replacements
		30104—Oral supplement liquids
		30105—Oral supplement powders
		32101—Infant formula
		32102—Human breast milk
		32103—Toddler formula, milk based
		Excluded major codes were:
		11—Non-alcoholic beverages,
		30—Special dietary foods
		33—Dietary supplements.
DED_food + all beverages_	All solid and liquid foods and all beverages including water	All food codes included in DED_food_ and DED_food + dairy beverages_ are included with the below additional codes;
		11—Non-alcoholic beverages
		11805—Other beverages (e.g., probiotics)
		27303005—Ice confection drink, crushed ice with fruit-based flavored syrup (25% apple juice)
		27303006—Ice confection, 97% fruit juice, tropical (apple, mango, passionfruit and banana)
		32401—Infant fruit juices
		Excluded major codes were:
		30—Special dietary foods
		33—Dietary supplements.

^1^ Some AUSNUT 2007 food codes which would be relevant for calculating energy density were not used in DED calculations because no children in the Melbourne InFANT Program consumed these foods or beverages; ^2^ Exceptions for specific food groups are noted.
